# The state of the art in therapeutic administration of botulinum toxin in children with cerebral palsy: an integrative review

**DOI:** 10.1590/1984-0462/2024/42/2023093

**Published:** 2024-03-25

**Authors:** Sandro Rachevsky Dorf, Adriana Rodrigues Fonseca, Flávio Roberto Sztajnbok, Thiffany Rodrigues Delfino de Oliveira, Linamara Rizzo Basttistella

**Affiliations:** a Instituto de Puericultura e Pediatria Martagão Gesteira, Universidade Federal do Rio de Janeiro, Rio de Janeiro, RJ, Brasil.; b Universidade Federal do Rio de Janeiro, Rio de Janeiro, RJ, Brasil.; c Hospital das Clínicas, Universidade de São Paulo, São Paulo, SP, Brasil.

**Keywords:** Botulinum toxin, Cerebral palsy, Spasticity, Rehabilitation, Toxina botulínica, Paralisia cerebral, Espasticidade, Reabilitação

## Abstract

**Objective:**

To describe the current state of the art in the therapeutic administration of botulinum toxin with indications, efficacy, and safety profile for children and adolescents with cerebral palsy.

**Data source:**

An integrative review was conducted. The MEDLINE/PubMed database was searched twice within the last decade using distinct terms, and only studies written in the English language were included. The study population was limited to those aged 0–18 years. Articles that were duplicates or lacked sufficient methodology information were excluded.

**Data synthesis:**

We found 256 articles, of which 105 were included. Among the included studies, most were conducted in developed countries. Botulinum toxin demonstrated good safety and efficacy in reducing spasticity, particularly when administered by a multidisciplinary rehabilitation team. It is primarily utilized to improve gait and upper limb function, facilitate hygiene care, reduce pain, prevent musculoskeletal deformities, and even decrease sialorrhea in patients without a functional prognosis for walking.

**Conclusions:**

The administration of botulinum toxin is safe and efficacious, especially when combined with a multi-professional rehabilitation team approach, which increases the probability of functional improvement. It can also be beneficial for patients with significant functional impairments to help with daily care tasks, such as hygiene, dressing, and reducing sialorrhea. Pediatricians must be familiar with this treatment and its indications to attend to and refer patients promptly when necessary, and to exploit their neuroplasticity. Further research on this topic is required in developing countries.

## RESUMO

**Objetivo:**

Descrever o estado da arte em aplicação terapêutica de toxina botulínica com indicações, eficácia e perfil de segurança em crianças e adolescentes com paralisia cerebral.

**Fontes de dados:**

Realizada revisão integrativa através de busca na base de dados MEDLINE/PubMed em dois momentos nos últimos 10 anos, e termos distintos, em inglês, numa população entre 0 e 18 anos de idade. Excluiu-se artigos duplicados ou com informações insuficientes de metodologia.

**Síntese dos dados:**

256 artigos foram encontrados e 105 foram incluídos, sendo a maior parte realizados em países desenvolvidos. A toxina botulínica mostrou boa segurança e efetividade na redução da espasticidade, especialmente administrada por uma equipe de reabilitação multiprofissional, usada principalmente para: melhora da marcha e da função dos membros superiores, facilitação dos cuidados de higiene, analgesia e prevenção de deformidades musculoesqueléticas, além de redução da sialorreia, inclusive em pacientes sem prognóstico funcional de marcha.

**Conclusões:**

A aplicação de toxina botulínica foi efetiva e segura, principalmente quando atrelada a uma abordagem por equipe de reabilitação multiprofissional, o que aumenta as chances de melhora funcional. Mostrou-se benéfica também para pacientes com grandes comprometimentos funcionais para facilitar os seus cuidados diários em relação à higiene, colocar e tirar roupas e redução da sialorreia. O pediatra deve estar familiarizado com esse tratamento e suas indicações para atender e direcionar pacientes o mais breve possível quando indicado e aproveitar o máximo de neuroplasticidade. Há necessidade de investimentos em mais pesquisas sobre este tema em países em desenvolvimento.

## INTRODUCTION

Botulinum toxin (BTX) is a biologically derived medication naturally produced by *Clostridium botulinum*, an anaerobic bacterium that produces eight serological types of toxins. The most potent is type A (BTX-A) and, therefore, it is used clinically in Brazil. BTX is a laboratory-produced biological agent. It is a stable crystalline substance lyophilized in human albumin and supplied in a sterile vacuum vial for dilution in saline solution.^1^

Its action occurs selectively in the cholinergic peripheral nerve terminal, inhibiting the release of acetylcholine by cleaving SNAP 25 (essential protein for the coupling and release of acetylcholine from nerve terminal vesicles located within the nerve endings), which causes muscle denervation and subsequent paralysis. Typically, recovery from an intramuscular injection occurs within 12 weeks due to the growth and formation of new connections between nerve endings and terminal plates. This results in transient selective muscle relaxation for therapeutic purposes or to facilitate daily care. It is administered only to specific muscles and in controlled doses based on the child’s weight.^1^ Neuromuscular blockade with BTX offers the following advantages: it allows access to specific muscles, it has a sustainable and reversible effect, and it reduces the chances of systemic adverse effects (e.g., a condition similar to botulism with generalized muscle relaxation) when compared to other oral medications (e.g., baclofen or benzodiazepines) or injectables (e.g., intrathecal baclofen pump or intravenous benzodiazepines).^1,2^ Since the 1990s, BTX injections have been administered for an increasing number of indications in human therapy, and clinical research is adding new indications.^1^

BTX administration for cerebral palsy (CP) has proved to be of great therapeutic value in reducing spasticity, particularly in gait improvement, upper limb function enhancement, hygiene care facilitation, pain relief, and prevention of myo-articular deformities.^3,4^

Pediatricians must be familiar with this treatment and its indications to refer patients promptly when necessary and take the maximum advantage of their neuroplasticity. This study, as an integrative review, aimed to provide a comprehensive overview of the therapeutic administration of BTX in children and adolescents with CP aged 0–18 years. This review focused on the main indications, adjuvant therapies, efficacy for intended objectives (e.g., improving gait patterns, upper limb function, facilitating axillary and/or inguinal hygiene care, providing pain relief, and preventing musculoskeletal deformities), and safety profile.

## METHOD

In this study, we conducted an integrative review of the therapeutic administration of BTX in children and adolescents with CP. Two authors searched the Medical Literature Analysis and Retrieval System Online (MEDLINE/PubMed) database twice using specific terms. The first search was conducted on March 2, 2022 using the search terms botulinum toxin, cerebral palsy, spasticity and child rehabilitation, and Medical Subject Headings (MeSH) terms “BOTULINUM TOXIN” AND “CEREBRAL PALSY” and “CHILD” AND “REHABILITATION” for literature published in the last ten years, in English, and limited to the age group of 0–18 years. The second search was conducted on June 2, 2022 for articles published in the last decade in English with the MeSH terms ”BOTULINUM TOXIN” AND “CEREBRAL PALSY” AND “SPASTICITY” AND “REHABILITATION” using the following search filters: “(“botulinum toxins”[MeSH Terms] OR (“botulinum”[All Fields] AND “toxins”[All Fields]) OR “botulinum toxins”[All Fields] OR (“botulinum”[All Fields] AND “toxin”[All Fields]) OR “botulinum toxin”[All Fields]) AND (“cerebral palsy”[MeSH Terms] OR (“cerebral”[All Fields] AND “palsy”[All Fields]) OR “cerebral palsy”[All Fields]) AND (“muscle spasticity”[MeSH Terms] OR (“muscle”[All Fields] AND “spasticity”[All Fields]) OR “muscle spasticity”[All Fields] OR “spastic” [All Fields] OR “spasticity”[All Fields] OR “spastics”[All Fields] OR “spasticity”[All Fields]) AND (“rehabilitant”[All Fields] OR “rehabilitants”[All Fields] OR “rehabilitate” [All Fields] OR “rehabilitated”[All Fields] OR “rehabilitates”[All Fields] OR “rehabilitating”[All Fields] OR “rehabilitation”[MeSH Terms] OR “rehabilitation”[All Fields] OR “rehabilitations”[All Fields] OR “rehabilitative”[All Fields] OR “rehabilitation”[MeSH Subheading] OR “rehabilitations”[All Fields] OR “rehabilitational”[All Fields] OR “rehabilitator”[All Fields] OR “rehabilitators”[All Fields]) AND ((y_10[Filter]) AND (allchild[Filter]))”.

Three authors extracted the data and excluded articles that were duplicated or lacked sufficient information in their methodology description. Because it is not the proposal of an integrative review, no quality analysis of the articles was performed. However, studies with more consistent outcomes were highlighted in the discussion.

All articles were analyzed by two authors. Clinical trials were analyzed separately due to their stronger evidence compared to the other studies included in this integrative review.

## RESULTS

**Figure 1:**
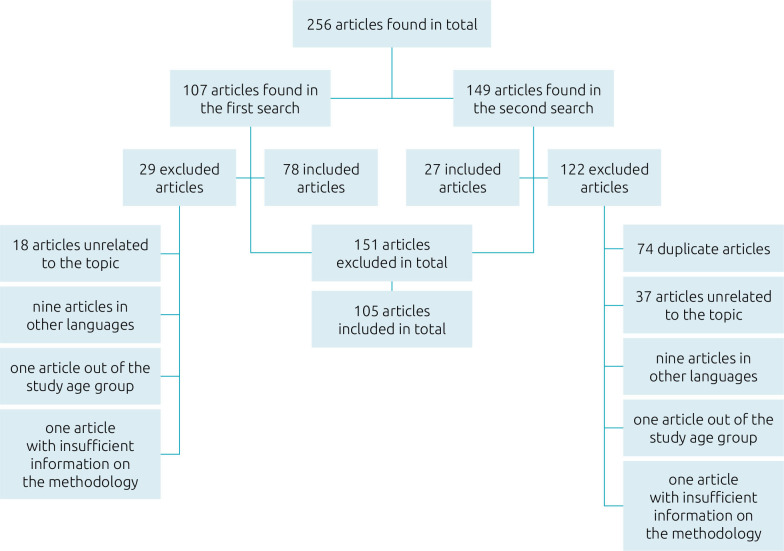
Organization chart on the data source to compose the integrative review.

The initial search yielded 78 articles, and the subsequent search added 27, resulting in 105 articles. All disagreements between the authors during the analysis were resolved by discussion until 100% consensus. Figure 1 shows the reasons for excluding the articles from the search. The age range of the participants in the studies (n=25,910) varied from one month to 23 years old (Figure 1).

Regarding the continent where the studies were conducted, the distribution was as follows: Europe (n=31; 29.5%), Asia (n=27; 25.7%), North America (n=17; 16.1%), Euro-Asia (Turkey) (n=15; 14.3%), Oceania (n=10; 9.5%), South America (n=3; 2.8%), and Africa (n=2; 1.9%).

As of the absence of relevant data in the included articles, three studies (2.9%) did not specify the age range, 36 (34.3%) did not indicate whether the patient was undergoing rehabilitation treatment, and 53 (50.5%) did not provide information on adverse effects (Tables 1–6).^4-105^

**Table 1:** Results: Gait improvement (Part 1).

SRN	Year (country)	Type	Sample (age)*	RP	Adverse events
5	2020 (Belgium)	CT	24 (7–9)	NI	NI
6	2020 (Turkey)	CT	241 (2–17)	POT	A
7	2020 (Spain)	CS	69 (2–18)	NI	NI
8	2020 (South Korea)	CT	29 (3–14)	NI	NI
9	2020 (Australia)	CT	40 (8–16)	NI	A
10	2019 (South Korea)	CT	591 (2–13)	P	NI
11	2019 (Turkey)	CT	118 (3–10)	POT	pain at the application site and bruising
12	2019 (Norway)	CT	1414 (5–8)	POT	NI
13	2019 (Turkey)	CT	30 (3–13)	P	redness at the application site
14	2019 (Egypt)	CT	60 (4–7)	P	A
15	2019 (USA)	CT	52 (1mo–17yrs)	NI	redness at the application site, erythema at the application site (face), mild dysphagia, dry mouth, and thick saliva
16	2019 (Turkey)	SR	153 (0–15)	P	pain and redness at the application site
17	2018 (Poland)	CT	60 (2–16)	P	NI
18	2018 (Brazil)	SR	111 (0–18)	P	temporary muscle weakness, dysesthesia, and pain at the injection site
19	2018 (USA)	CT	10 (2–12)	NI	NI
20	2018 (Japan)	CT	9 (4–8)	NI	NI
21	2017 (India)	CT	29 (2–7)	POT	fever associated with respiratory tract infection, and muscle weakness for a few days at the application site
22	2017 (Australia)	RC	17 (2–6)	P	NI
23	2017 (South Korea)	CT	144 (2–10)	NI	NI
24	2017 (South Korea)	CT	144 (2–10)	NI	urticaria and dysphonia in the Botox^®^ group
25	2017 (Sweden)	CT	40 (4–12)	P	A
26	2017 (Turkey)	CT	51 (3–17)	P	A
27	2016 (Turkey)	SR	893 (1–19)	NI	NI
28	2016 (Australia)	CT	42 (2–5)	P	flu-like symptoms, localized muscle pain, vomiting, and occasional gait instability

*Years. SRN: study reference number; RP: rehabilitation program; CT: clinical trial; NI: not informed; POT: physiotherapy and occupational therapy; A: absent; CS: case series; P: physiotherapy; SR: systematic review; RC: retrospective cohort.

**Table 2:** Results: Gait improvement (Part 2).

SRN	Year (country)	Type	Sample (age)*	RP	Adverse events
29	2016 (Belgium)	RC	53 (4–18)	P	NI
30	2016 (South Korea)	CT	25 (3–15)	P	NI
31	2016 (Australia)	PC	10 (6–16)	NI	NI
32	2016 (USA)	CT	241 (2–17)	P	temporary muscle weakness
33	2014 (Australia)	CT	6 (8–9)	P	A
34	2014 (Belgium)	CT	31 (3–18)	P	NI
35	2014 (China)	CT	37 (3–15)	P	NI
36	2014 (Turkey)	CT	33 (2–8)	P	A
37	2014 (Belgium)	CT	19 (3–18)	P	NI
38	2013 (Australia)	CT	15 (5–12)	P	NI
39	2012 (South Korea)	CT	40 (2–14)	NI	NI
40	2012 (South Korea)	CT	17 (2–9)	P	A
41	2022 (Czech Republic)	CT	370 (2–17)	POT	seizure in patient with epilepsy, constipation and muscle weakness
42	2022 (USA)	CT	381 (2–17)	P	upper respiratory tract infections, followed by fever and cough
43	2021 (USA)	CSR	1508 (0–19)	P	NI
44	2021 (Poland)	NSR	945 (NI)	POT	respiratory tract infection, bronchitis, pharyngitis, asthma, muscle weakness, urinary incontinence, falls, seizures, temporary low-grade fever, and pain at the application site
45	2019 (Turkey)	SR	153 (11mo–15yrs)	P	pain, redness, and dysesthesia at the application site, and muscle weakness
46	2018 (Canada)	Q	15 (5–17)	NI	NI
47	2016 (China)	CT	4 (5–8)	POT	NI
48	2014 (Poland)	CT	41 (2–15)	P	A
49	2014 (South Korea)	CT	25 (2–6)	NI	NI
50	2013 (Brazil)	CT	14 (2–18)	P	A
51	2013 (China)	CT	244 (1–23)	P	NI

*Years. SRN: study reference number; RP: rehabilitation program; RC: retrospective cohort; P: physiotherapy; NI: not informed; CT: clinical trial; PC: prospective cohort; A: absent; POT: physiotherapy and occupational therapy; CSR: Cochrane systematic review; NSR: non-systematic review; SR: systematic review; Q: qualitative.

**Table 3:** Results: Upper limbs function improvement.

SRN	Year (country)	Type	Sample (age)*	RP	Adverse events
52	2021 (USA)	CT	372 (2–17)	POT	pain at the application site
53	2020 (Turkey)	CT	210 (2–17)	OT	Nausea
7	2020 (Spain)	CS	69 (2–18)	NI	NI
54	2020 (Saudi Arabia)	CT	64 (6–10)	P	NI
12	2019 (Norway)	CT	1414 (5–8)	POT	NI
15	2019 (USA)	CT	52 (1mo–17yrs)	NI	redness at the application site, erythema at the application site (face), mild dysphagia, dry mouth, and thick saliva
27	2016 (Turkey)	SR	893 (1–19)	NI	NI
31	2016 (Australia)	PC	10 (6–16)	NI	NI
55	2015 (China)	CT	12 (3–12)	OT	A
56	2015 (Sweden)	CT	20 (1.5–10)	OT	A
57	2015 (Netherlands)	CT	35 (4–9)	POT	A
58	2014 (Italy)	CT	27 (3–9)	P	A
41	2022 (Czech Republic)	CT	370 (2–17)	POT	seizure in patient with epilepsy, constipation, and muscle weakness
44	2021 (Poland)	NSR	945 (NI)	POT	respiratory tract infection, bronchitis, pharyngitis, asthma, muscle weakness, urinary incontinence, falls, seizures, temporary low-grade fever, and pain at the application site
59	2020 (Sweden)	CS	25 (1–23)	POT	NI
60	2019 (Sweden)	CT	20 (1–4)	OT	NI
46	2018 (Canada)	Q	15 (5–17)	NI	NI
50	2013 (Brazil)	CT	14 (2–18)	P	A

*Years. SRN: study reference number; RP: rehabilitation program; CT: clinical trial; POT: physiotherapy and occupational therapy; OT: occupational therapy; CS: case series; NI: not informed; P: physiotherapy; SR: systematic review; PC: prospective cohort; A: absent; NSR: non-systematic review; Q: qualitative.

**Table 4:** Results: deformities prevention, hygiene improvement, and pain relief.

Deformities prevention					
SRN	Year (country)	Type	Sample (age)*	RP	Adverse events
61	2018 (Turkey)	CT	17 (4–8)	P	NI
17	2018 (Poland)	CT	60 (2–16)	P	NI
19	2018 (USA)	CT	10 (2–12)	NI	NI
62	2017 (South Korea)	CT	144 (2–10)	NI	urticaria and dysphonia in the Botox^®^ group
26	2017 (Turkey)	CT	51 (3–17)	P	A
41	2022 (Czech Republic)	CT	370 (2–17)	POT	seizure in patient with epilepsy, constipation and muscle weakness
63	2021 (South Korea)	CT	20 (2–10)	NI	NI
14	2019 (Egypt)	CT	60 (4–7)	P	NI
46	2018 (Canada)	Q	15 (5–17)	NI	NI
48	2014 (Poland)	CT	41 (2–15)	P	A
49	2014 (South Korea)	CT	25 (2–6)	NI	NI
50	2013 (Brazil)	CT	14 (2–18)	P	A
64	2014 (Australia)	CT	41 (2–16)	POT	pain and bruising at the application site
**Hygiene improvement**					
7	2020 (Spain)	CS	69 (2–18)	NI	NI
12	2019 (Norway)	CT	1414 (5–8)	POT	NI
15	2019 (USA)	CT	52 (1mo–17yrs)	NI	redness at the application site, erythema at the application site (face), mild dysphagia, dry mouth, and thick saliva
64	2014 (Australia)	CT	41 (2–16)	POT	pain and bruising at the application site
46	2018 (Canada)	Q	15 (5–17)	NI	NI
50	2013 (Brazil)	CT	14 (2–18)	P	A
**Pain relief**					
65	2021 (Lithuania)	SR	644 (0–18)	POT	A
31	2016 (Australia)	PC	10 (6–16)	NI	NI
64	2014 (Australia)	CT	41 (2–16)	POT	pain and bruising at the application site
15	2019 (USA)	RC	52 (1mo–18yrs)	NI	redness at the application site, erythema at the application site (face), mild dysphagia, dry mouth, and thick saliva
46	2018 (Canada)	Q	15 (5–17)	NI	NI

*Years. SRN: study reference number; RP: rehabilitation program; CT: clinical trial; P: physiotherapy; NI: not informed; A: absent; POT: physiotherapy and occupational therapy; Q: qualitative; CS: case series; SR: systematic review; PC: prospective cohort; RC: retrospective cohort.

**Table 5:** Safety and effects assessments of Botulinum toxin injections and adjunct therapies effects and Botulinum toxin injections effects.

Safety and effects assessments of BTX injections					
SRN	Year (country)	Type	Sample (age)*	RP	Adverse events
66	2021 (Thailand)	RC	1405 (0–18)	NI	A
67	2021 (Turkey)	SC	60 (0–18)	POT	A
68	2020 (Turkey)	CT	33 (3–7)	NI	NI
69	2020 (Japan)	CT	24 (2–11)	P	NI
70	2019 (USA)	CT	256 (1–21)	NI	NI
71	2018 (Netherlands)	O	65 (4–12)	P	A
72	2017 (USA)	CR	2 (6–11)	A	A
73	2016 (Turkey)	CS	24 (3–12)	NI	NI
74	2016 (Turkey)	CT	25 (4–22)	POT	excessive weakness at the application site
75	2015 (Australia)	CT	41 (2–16)	NI	pain at the application site and temporary muscle weakness
76	2015 (USA)	NSR	98 (3–11)	NI	temporary muscle weakness, transient pain, irritability, nausea, vomiting, fatigue, increased seizure episodes, increased salivation, slower speech, transient low-grade fever, and blister-like lesion at the application site
77	2021 (Sweden)	CT	8817 (4–19)	NI	NI
78	2021 (USA)	SR	761 (0–2)	POT	local or generalized weakness, pain at the application site, bruising, and vomiting
79	2019 (France)	NSR	NI	NI	pseudo influenza, rash, pain, cramps, and bruising at the application site
17	2018 (Poland)	CT	60 (2–16)	NI	NI
80	2016 (China)	CR	1 (17)	POT	NI
81	2022 (China)	CT	91 (2–10)	NI	NI
**Adjunct therapies effects assessment and BTX injections effects**					
82	2019 (France)	SR	662 (3–13)	POT	pain, skin irritation, and muscle atrophy associated with plaster immobilization
83	2018 (Netherlands)	CT	65 (4–12)	P	A
84	2018 (USA)	CT	65 (2–17)	POT	NI
85	2017 (Italy)	CT	10 (3–14)	P	NI
86	2014 (South Korea)	CT	38 (2–14)	P	NI
87	2014 (Australia)	CT	30 (4–14)	P	NI
88	2013 (India)	CT	36 (2–8)	P	NI
89	2019 (South Korea)	SR	1264 (1–9)	POT	NI

*Years. BTX: Botulinum toxin; SRN: study reference number; RP: rehabilitation program; RC: retrospective cohort; NI: not informed; A: absent; SC: specialists’ consensus; POT: physiotherapy and occupational therapy; CT: clinical trial; O: others; P: physiotherapy; CR: case report; CS: case series; NSR: non-systematic review; SR: systematic review.

**Table 6:** Muscle structure effects, cost-effectiveness assessment, and recommended procedure techniques.

Muscle structure effects					
SRN	Year (country)	Type	Sample (age)*	RP	Adverse events
90	2021 (South Korea)	CT	15 (2–12)	NI	NI
91	2019 (South Korea)	CT	14 (2–10)	P	discomfort, decline in general condition, headache, dry mouth, pain at the injection site, viral infection, dizziness, back pain, and fatigue
92	2019 (Belgium)	CT	67 (7–11)	P	NI
93	2018 (Australia)	PC	11 (5–13)	NI	A
94	2017 (Turkey)	PC	12 (6–14)	NI	A
95	2014 (Brazil)	SR	480 (1.5–16)	POT	NI
96	2014 (South Korea)	PC	13 (4–8)	P	A
97	2022 (Belgium)	PC	26 (2–9)	P	A
**Cost-effectiveness assessment**					
4	2020 (Netherlands)	CES	60 (NI)	P	NI
**Recommended procedural techniques**					
98	2018 (USA)	SC	307 (NI)	NI	NI
99	2018 (Turkey)	CT	25 (3–16)	P	A
100	2018 (USA)	CT	14 (4–13)	NI	A
101	2018 (USA)	RC	249 (5–9)	NI	NI
102	2017 (Thailand)	CS	116 (30–46)	NI	NI
103	2016 (Netherlands)	PC	75 (4–18)	NI	NI
104	2013 (Netherlands)	CT	NI	NI	NI
105	2020 (France)	CT	59 (5–11)	NI	NI

*Years. SRN: study reference number; RP: rehabilitation program; CT: clinical trial; NI: not informed; P: physiotherapy; PC: prospective cohort; A: absent; SR: systematic review; POT: physiotherapy and occupational therapy; CES: cost-effectiveness study; SC: specialists’ consensus; RC: retrospective cohort; CS: case series.

Concerning clinical trials, 22 (31.4%) were conducted in Asia, 20 (28.6%) in Europe, nine (12.9%) in North America, nine (12.9%) in Euro-Asia (specifically Turkey), seven (10.0%) in Oceania, two (2.9%) in Africa, and one (1.4%) in South America.

Referring to systematic reviews, three (30%) originated from Euro-Asia (Turkey), two (20%) from Europe, two (20%) from North America, two (20%) from South America, and one (10%) was from Asia (Tables 1–6)^4-105^

The description of the objectives of the BTX administration included in the studies was categorized into different domains: improvement of gait (n=71; 67.6%), enhancement of upper limb motor function (n=28; 26.7%), facilitation of axillary and inguinal hygiene care (n=15; 14.3%), reduction of sialorrhea (n=14; 13.3%), prevention of musculoskeletal deformities (n=9; 8.6%), and improvement of pain (n=5; 4.8%). Furthermore, a significant portion of the studies aimed to cover more than one domain (n=30; 28.6%), and three studies (2.9%) lacked data on their objectives. Notably, 92.4% (n=97) of the studies achieved the proposed objective.

The most commonly used BTX in the studies was Botox^®^ (n=73; 69.5%), followed by Dysport^®^ (n=27; 25.7%), Xeomin^®^ (n=8; 7.6%), Myobloc^®^ (n=4; 3.8%), Medytox^®^/Botulift^®^ (n=3; 2.9%), Prosigne^®^ (n=1; 0.9%), and Botulim^®^/Botulax^®^ (n=1; 0.9%); and 4 (3.8%) did not specify which BTX was administered. It is important to note that many studies used more than one type of BTX.

Regarding the type of BTX adopted in clinical trials (n=70), the majority exclusively used Botox^®^ (n=36; 51,4%), while Dysport^®^ was the second most commonly used (n=10; 14.3%).

Regarding the number of administrations, most studies (n=75; 71.4%) performed up to two administrations, followed by five or more (n=7; 6.7%), up to three administrations (n=5; 4.8%), and up to four (n=2; 1.9%); and 16 studies (15.2%) did not report the number of administrations performed.

Regarding orthoses, 39 studies (37.1%) utilized this device; however, most did not report this information (n=65; 31.9%).

Considering the effective improvement in gait outcomes, this result was achieved in a clinical trial conducted by Dursun et al.^6^ The trial evaluated the efficacy of Dysport^®^ injections in 241 patients aged 2–17 years. Kim et al. carried out a clinical trial with 29 patients between the ages of 3–14 years, demonstrating an improvement in gait performance compared to knee flexion without altering the muscular structure.^8^ In another clinical trial conducted by Wesseling M involving 24 patients aged 7–9 years, there was no deterioration in gait stability after the administration of BTX.^5^ Valentine J carried out a clinical trial involving 40 children and adolescents (8–16 years old) with CP classified in Gross Motor Function Classification System (GMFCS) level II, which demonstrated an increase in gait functionality after the administration of BTX.^9^ Hastings-Ison et al. performed a clinical trial with 42 children aged 2–5 years to compare the efficacy of one annual Botox^®^ administration versus three annual administrations for the treatment of spastic dynamic equinus. The annual administration demonstrated the same level of efficacy as the three annual administrations. This study found that 36.3% of the participants experienced moderate adverse effects, including flu-like symptoms, localized muscle pain, vomiting, and occasional gait instability.^28^

Regarding the functional improvement outcome of the upper limbs, a favorable result was achieved with a good safety profile in a clinical trial conducted by Delgado et al. with 210 patients between 2–17 years of age with the administration of Dysport^®^.^53^ There were good functional results for BTX administration in upper limbs with the association of functional electrical stimulation in a clinical trial conducted by Elnaggar et al.^54^

In the clinical trial by Lee et al., the administration of Meditoxin^®^ in the hips of CP children to prevent dislocation yielded positive results.^63^ Regarding the prevention of equinus deformity, Chang et al. conducted a clinical trial involving 144 children aged 2–10 years who received two types of BTX toxin: Botulax^®^ (letibotulinumtoxinA) and Botox^®^ (onabotulinumtoxinA), with improvement of gait and prevention of equinus deformity in both types of BTX. There were records of two patients (2.86%) who experienced adverse effects due to the administration of Botox^®^, including urticaria and short-term dysphonia.^24^

In a systematic review by Almina et al., the administration of BTX for spastic hips reduced pain. It facilitated daily care for non-ambulatory children with GMFCS levels IV and V, who could not walk.^65^ Botox^®^ and Dysport^®^ were used in the study.

The BTX administration program, provided by the public health system of Vale do Jequitinhonha, Brazil, effectively improved gait and functional independence for daily activities. This conclusion was drawn from a clinical trial conducted by Silva et at. involving 14 patients aged 2–18 years.^50^

The study conducted by Bussmann et al., which evaluated the cost-effectiveness of BTX in 60 children with walking capacity, found that BTX was efficacious when administered by specialist doctors in physical medicine and rehabilitation and/or trained neurologists.^4^

Systematic outpatient treatment with BTX injections, combined with physiotherapy, occupational therapy, plaster, and/or orthoses associated with rehabilitation team treatment, has been recommended in several studies to achieve the set objectives successfully.^3,4,45,59,82,60^ Hareb et al. conducted a non-systematic review. They reported that the administration of BTX is well-supported in the literature, especially for spasticity in children over 2 years old, requiring a multidisciplinary approach. The following BTXs were included in the studies in this review: Botox^®^, Dysport^®^, and Xeomin^®^. There were reports of adverse effects such as pseudo-influenza, skin rashes, pain, cramps, and bruises at the administration site.^79^

Yi et al. conducted a clinical trial with 14 children aged 2–10 years with hemiplegia who had equinus gait on the affected side to evaluate whether the repeated administrations of Botox^®^ could impact the growth and strength of the gastrocnemius muscle fibers. The study reported no significant difference in muscle structure due to successive administration. There were reports of possible adverse effects during administration, including discomfort, general weakness, headache, dry mouth, pain at the injection site, viral infection, dizziness, back pain, and fatigue.^91^

Shoval et al. conducted a clinical trial with 52 patients aged one month to 17 years to evaluate the administration of Botox^®^ in salivary glands and multiple muscle segments to treat sialorrhea and axillary and inguinal hygiene. The majority of patients had severe functional CP sequelae. Consequently, there was a decrease in drooling and an improvement in upper limb function, gait, and axillary and inguinal hygiene. Adverse effects were observed in 4% of patients who received Botox^®^, in the salivary glands, and 7% of those who received BTX, in the glands and muscles. The most severe adverse effects included redness at the administration site lasting for one day, dry mouth for two weeks, and mild difficulty swallowing thick saliva for two months.^15^

Juneja et al. conducted a clinical trial involving 29 children aged 2–7 years who received Botox^®^ injections and an intensive rehabilitation program, which improved the gait pattern. Adverse effects were fever caused by mild upper respiratory tract infection (10.3%) and temporary muscular weakness (6.9%).^21^ Yana et al. conducted a systematic review on the efficacy of Botox^®^ and/or Dysport^®^ injections in the lower limbs of spastic children with CP. The study reported that the administration improved spasticity and range of motion when combined with physiotherapy. However, the results of this systematic review were inconclusive regarding whether the combination of BTX and physiotherapy is more effective than isolated physiotherapy at improving motor function. The adverse effects described were pain at the administration site, muscle weakness, and localized dysesthesia.^45^

Regarding the adverse effects reported in clinical trials, 13 participants (12.4%) experienced systemic adverse effects. Out of these, seven (53.8%) were treated with Botox^®^, two (15.4%) with both Botox^®^ and Dysport^®^, one (7.7%) with Botulax^®^ and Botox^®^, one (7.7%) was treated with Botox^®^, Dysport^®^, and Xeomin^®^, and one (7.7%) did not specify which BTX was used (Tables 1-6).^4-105^

Regarding safety and efficacy, Ploypetch et al. conducted a medical chart review on the adverse effects of combined multilevel chemical blocks of Botox^®^ and 5% Phenol for spasticity at a university medical center in the United States. The study included 98 children aged 3–11 years who received at least one multilevel chemical block, resulting in a total of 146 administrations. The most frequent adverse effect was temporary muscle weakness, which occurred in 11.0% of cases and was accompanied by a higher incidence of falls, at 3.0%. The incidence of muscle weakness was significantly higher in the group that administered only Botox^®^ (20.0%) compared to the group that combined the administration of Botox^®^ and 5% Phenol (7.6%). Pain at the administration site (4.8%) and local blister (0.7%) only occurred in the group that performed combined administration. Other less frequent adverse effects were observed, including irritability (4.1%), nausea (4.1%), vomiting (4.1%), fatigue (4.1%), increased frequency of seizures (4.1%), sialorrhea (4.1%), slower speech (0.7%), and temporary low-grade fever (0.7%).^76^ In a clinical trial conducted by Kaňovský et al. involving 370 patients aged 2–17 years, the administration of Xeomin^®^ was safe and efficacious for spastic upper and lower limbs. There were reports of adverse effects in 4.3% of cases, including muscle weakness (0.3%), seizure in a patient with epilepsy (0.3%), and constipation (0.3%).^41^ In a clinical trial conducted by Dimitrova et al. involving 381 patients aged 2–17 years, physiotherapy was associated with significantly reduced spasticity and improved lower limb movement. Adverse effects were observed in 2.8% of cases, including mild to moderate exacerbation of seizures, which were more frequent in the group that received a dose of 8 U/K, but similar to the placebo group.^42^ Ayala et al. conducted a systematic review of interventions for infants with a confirmed diagnosis of CP suffering from spasticity. In cases of moderate to severe spasticity, they recommended the administration of BTX for this age group, as it positively impacts functionality and social participation despite the paucity of evidence. In this review, the authors observed several adverse effects, including muscle weakness, injection site pain, bruising, and vomiting.^78^ Bonikowski and Sławek conducted a non-systematic review to compare the safety and efficacy of Botox^®^, Xeomin^®^, and Dysport^®^, taking into consideration their pre-administration preparation. These treatments were safe and efficacious against both upper and lower limb hypertonia. However, some adverse effects have been reported, including respiratory tract infections, bronchitis, pharyngitis, asthma, muscle weakness, urinary incontinence, falls, seizures, temporary low-grade fever, and pain at the injection site. It is important to note that these adverse effects were temporary and had a low incidence rate.^44^

## DISCUSSION

The integrative review method enables the synthesis of knowledge and the incorporation of relevant study results into practice. It is the most thorough methodological approach among reviews, as it permits the inclusion of both experimental and non-experimental studies, thereby providing a comprehensive understanding of the phenomenon under consideration. This method combines theoretical and empirical literature data for a variety of purposes, which includes defining concepts, reviewing theories and evidence, and analyzing methodological problems related to a particular topic. The extensive sample and variety of proposals should generate a coherent and comprehensible overview of intricate concepts, theories, or health issues. Since 1980, the integrative review has been reported in the literature as a research method.^106,107^

It is extremely relevant the fact that it is a broad review on the subject, being the first integrative review on this topic worldwide on the therapeutic administration of BTX in children and adolescents with CP, according to PubMed. Furthermore, there were no significant study limitations and there was no conflict of interests by the authors, particularly with the pharmaceutical industry.

The methodology employed in this type of review enables the inclusion of more articles than in a systematic review. All 105 articles included demonstrated that BTX is safe and efficacious in treating spasticity, particularly when administered in a multi-professional rehabilitation team approach. This approach increases the likelihood of functional improvement in both gait and voluntary activities involving the upper limbs. However, it can be beneficial for patients who do not have a prognosis for walking or active movement of their upper limbs to facilitate their daily care in terms of hygiene, dressing and undressing, and reducing sialorrhea.

Most studies were conducted in Europe, Asia, and North America. Only 1.9% of the participants were from Africa, which may be attributed to socioeconomic challenges and limited access to medication. This highlights the urgent need for BTX administration research funding in developing countries.

The primary outcome was an improvement in gait, followed by an improvement in upper limb motor skills and in daily care, including axillary and inguinal hygiene. This demonstrates the vast therapeutic potential of this medication in children and adolescents with CP, even in patients without a functional prognosis for gait.

Most clinical trials (51.4%) were exclusively conducted with Botox^®^, suggesting that this BTX is the most commonly administered.

The articles highlighted the improvement in gait^5,6,8^ and upper limb^53^ functions due to BTX administration. These findings demonstrate the efficacy of BTX in these areas and its safety in terms of adverse effects. Ideally, the administration should be performed by medical specialists in physical medicine and rehabilitation (physiatry) and/or neurologists^106^ affiliated with a multi-professional rehabilitation program.^50^

The fact that 50.5% of articles lack information on adverse effects is extremely relevant, as such data is crucial for the clinical administration of BTX in terms of safety and effectiveness. There were reports of systemic adverse effects in 12.4% of clinical trials; however, no deaths were directly related to the administration of BTX. This reaffirms the safety of BTX administration in children with CP. The fact that Botox^®^ is the most commonly administered BTX in clinical trials justifies that 53.8% of clinical trials with systemic adverse effects involved Botox^®^. In 34.3% of the articles, no data was available on rehabilitation therapies administered concurrently with BTX. The effectiveness of BTX administration depends on multidisciplinary rehabilitation treatment.^106^

Our study has some limitations: the search for only the last ten years, the fact that only articles in English were analyzed, and no database other than MEDLINE/PubMed was used in the search for articles.

The administration of BTX for spasticity in children with CP is efficacious and safe. This is particularly true when combined with a multidisciplinary rehabilitation team approach, which increases the likelihood of functional improvement in both gait and voluntary upper limb movement. However, patients who do not have a prognosis of walking or active movement of their upper limbs can still benefit from using applications that facilitate their daily care, such as hygiene, dressing and undressing, and reducing sialorrhea, all these indications with safety and effectivity. Experienced and well-trained doctors, typically found in rehabilitation centers, physiatrists, and neuro-pediatricians, safely and effectively perform chemical blockade using BTX.
